# Genome-Wide Analysis and Expression Profile of Superoxide Dismutase (SOD) Gene Family in Rapeseed (*Brassica napus* L.) under Different Hormones and Abiotic Stress Conditions

**DOI:** 10.3390/antiox10081182

**Published:** 2021-07-25

**Authors:** Wei Su, Ali Raza, Ang Gao, Ziqi Jia, Yi Zhang, Muhammad Azhar Hussain, Sundas Saher Mehmood, Yong Cheng, Yan Lv, Xiling Zou

**Affiliations:** Key Lab of Biology and Genetic Improvement of Oil Crops, Oil Crops Research Institute, Chinese Academy of Agricultural Sciences (CAAS), Wuhan 430062, China; 82101181063@caas.cn (W.S.); alirazamughal143@gmail.com (A.R.); 1696555087@163.com (A.G.); jiaziqi0507@163.com (Z.J.); zhangyi20210615@163.com (Y.Z.); azharhussain301@gmail.com (M.A.H.); snookas.saher90@gmail.com (S.S.M.); chengyong@caas.cn (Y.C.)

**Keywords:** abiotic stress, antioxidant defense systems, gene ontology, miRNA, phytohormones, 3D structures

## Abstract

Superoxide dismutase (SOD) is an important enzyme that acts as the first line of protection in the plant antioxidant defense system, involved in eliminating reactive oxygen species (ROS) under harsh environmental conditions. Nevertheless, the *SOD* gene family was yet to be reported in rapeseed (*Brassica napus* L.). Thus, a genome-wide investigation was carried out to identify the rapeseed *SOD* genes. The present study recognized 31 *BnSOD* genes in the rapeseed genome, including 14 *BnCSDs*, 11 *BnFSDs*, and six *BnMSDs*. Phylogenetic analysis revealed that *SOD* genes from rapeseed and other closely related plant species were clustered into three groups based on the binding domain with high bootstrap values. The systemic analysis exposed that *BnSODs* experienced segmental duplications. Gene structure and motif analysis specified that most of the *BnSOD* genes displayed a relatively well-maintained exon–intron and motif configuration within the same group. Moreover, we identified five hormones and four stress- and several light-responsive *cis*-elements in the promoters of *BnSODs*. Thirty putative bna-miRNAs from seven families were also predicted, targeting 13 *BnSODs*. Gene ontology annotation outcomes confirm the *BnSODs* role under different stress stimuli, cellular oxidant detoxification processes, metal ion binding activities, SOD activity, and different cellular components. Twelve *BnSOD* genes exhibited higher expression profiles in numerous developmental tissues, i.e., root, leaf, stem, and silique. The qRT-PCR based expression profiling showed that eight genes (*BnCSD1, BnCSD3, BnCSD14, BnFSD4, BnFSD5, BnFSD6, BnMSD2,* and *BnMSD10*) were significantly up-regulated under different hormones (ABA, GA, IAA, and KT) and abiotic stress (salinity, cold, waterlogging, and drought) treatments. The predicted 3D structures discovered comparable conserved BnSOD protein structures. In short, our findings deliver a foundation for additional functional investigations on the *BnSOD* genes in rapeseed breeding programs.

## 1. Introduction

Several environmental cues, including abiotic and biotic traumas, are considered key influences affecting the plants’ productivity [1,2]. Under stressful conditions, the plant amends its homeostatic apparatus by developing an increased reactive oxygen species (ROS) in plant cells. Usually, ROS overproduction results in several molecular and cellular damages, and ultimately programmed cell death [3,4]. ROS such as superoxide anion, hydrogen peroxide, hydroxyl radical, perhydroxyl radicals, alkoxy radicals, peroxy radicals, singlet oxygen, and organic hydroperoxide, are considered to be major signaling molecules, regulating abiotic and biotic stress responses, and also participate in plant productivity [3,4]. Mainly, ROS are formed in the apoplast, mitochondria, plasma membrane, chloroplast, peroxisomes, endoplasmic reticulum, and cell walls [3,4]. Therefore, to manage ROS noxiousness, plants have established well-organized and composite antioxidant defense systems, including numerous non-enzymatic and enzymatic antioxidants [3,4]. 

Among numerous antioxidant enzymes, superoxide dismutase (SOD; EC 1.15.1.1), a set of metalloenzymes, largely exists in alive organisms. SOD acts as the first line of ROS scavenging and plays a crucial role in plants’ physio-biochemical procedures to manage environmental cues [3]. Findings revealed that SOD catalyzes the superoxide radicals’ dismutation into oxygen and hydrogen peroxide via disproportionation, and protects the plant cells from oxidative injury [5,6]. According to metal cofactors, plant SODs are largely characterized into four major groups such as copper-zinc SOD (Cu/ZnSOD), iron SOD (FeSOD), manganese SOD (MnSOD), and nickel SOD (NiSOD) [7,8]. Among them, NiSOD mainly exists in Streptomyces, cyanobacteria, and marine life, and is yet to be described in plants [9]. However, FeSODs and MnSODs are primarily extant in lower plants, while Cu/ZnSODs exist in higher plants [10,11]. These SODs are widely distributed in various cell organs. For instance, Cu/ZnSODs are amply present, and mostly distributed in chloroplasts, cytoplasm, and peroxisomes [12]. FeSOD is distributed in the chloroplasts, and MnSODs are distributed in mitochondria and peroxisomes [8,13].

Recently, numerous investigations have revealed that the transcript level of the plant *SODs* gene responds to several environmental cues and helps plants cope with harsh environmental conditions. For instance, the increased SOD activity helps plants to show resistance to salinity and drought stress in *Brassica juncea* plants [14], temperature-induced oxidative damage in *Acutodesmus dimorphus* [15], cold-induced oxidative damage in tomato (*Solanum lycopersicum*) [16], etc. Moreover, the expression of the *Cu/ZnSOD* gene was persuaded by the copper-nanoparticle application in cucumber (*Cucumis sativus*) [17]. Under cold stress, trehalose application modulated the expression profile of the *Cu/ZnSOD* gene in tomato plants [16]. In a recent study, the overexpression of *SikCuZnSOD3* enhances tolerance to cold, drought, and salinity stresses in cotton (*Gossypium hirsutum*) [18]. Under water deficit conditions, wheat (*Triticum aestivum*) plants showed a higher expression of different antioxidant encoding genes, including *MnSOD* [19]. Likewise, under 1-methylcyclopropene (1-MCP) supplementation, apple (*Malus×domestica* Borkh.) fruits showed a higher expression of *Cu/ZnSODs, MnSODs,* and *FeSODs* [20]. In short, these findings showed that improved SOD activity and higher expression of SOD-encoding genes can contribute to plant tolerance to multiple stresses. 

Additionally, earlier investigations recommended that miRNA-mediated regulation of ROS-accompanying genes is crucial for plant productivity [21,22] and stress resistance [23,24,25,26]. For example, miR398 targets two *Cu/ZnSOD* genes in *Arabidopsis thaliana* [27]. In another study, 20 miRNAs are found to be targeting 14 cotton *SOD* genes at 33 predicted sites [28]. Moreover, ghr-miR414c, ghr-miR7267, m0081, m0166, and m0362 are found to play a vital role in cotton fiber variation and progress [29,30], and ghr-miR3 controls the transcript level of targeted genes throughout cotton somatic embryogenesis [31]. In *A. thaliana*, two *Cu/ZnSOD* genes (*CSD1* and *CSD2*) are induced via the down-regulation of miR398, which helps plants to enhance tolerance to oxidative injury [32]. These discoveries stated that miRNAs might play active roles against environmental cues and plant development through modifying the *SOD* genes.

Rapeseed (*Brassica napus* L.) is the second imperative oilseed crop and possesses a complex genome. Several abiotic stresses significantly limit rapeseed productivity [33,34,35,36]. To date, *SOD* family genes have not been reported in rapeseed. Thus, in the present study, we performed a genome-wide analysis to identify *SOD* genes in the rapeseed genome. Additionally, their phylogenetic relationships, synteny analysis, gene structures, conserved motifs, *cis*-elements, miRNA predictions, functional annotations, and 3D structures have been characterized. Moreover, the expression profile in numerous tissues/organs and under numerous hormone and abiotic stress conditions have been extensively appraised, which deeply boosted our understanding of the *SOD* genes in rapeseed.

## 2. Materials and Methods

### 2.1. Identification and Characterization of SOD Genes in Rapeseed

According to our recent study [37], we used two methods to identify *SOD* genes in the *B. napus* genome, i.e., BLASTP (protein blast) and the Hidden Markov Model (HMM) [37]. The *B. napus* genome sequence was downloaded from the BnPIR database (Available online: http://cbi.hzau.edu.cn/bnapus/index.php, accessed on 1 April 2021) [38]. For BLASTP, we used eight *A. thaliana* SODs (AT1G08830.1/*AtCSD1*, AT2G28190.1/*AtCSD2*, AT5G18100.1/*AtCSD3*, AT4G25100.1/*AtFSD1*, AT5G51100.1/*AtFSD2*, AT5G23310.1/*AtFSD3*, AT3G10920.1/*AtMSD1*, and AT3G56350.1/*AtMSD2*) amino acid sequences as a query with an e-value set to 1e^−5^. The amino acid sequences of eight *AtSODs* were obtained from the TAIR *Arabidopsis* genome database (Available online: http://www.arabidopsis.org/, accessed on 1 April 2021). [39]. Further, the local HMMER 3.1 web server (Available online: http://www.hmmer.org/, accessed on 1 April 2021) [40] was used to search the *SOD* genes with default parameters. Then, the HMM file of the Sod_Cu (PF00080.21) and Sod_Fe_C (PF02777.19) having *SOD* genes were downloaded from the Pfam protein domain database (Available online: http://pfam.xfam.org/, accessed on 1 April 2021) [41]. Finally, 31 *BnSOD* genes were identified by combining the two methods in the rapeseed genome. Moreover, we identified *SOD* genes in different plant species, such as *Brassica rapa*, and *Brassica oleracea*, with the genome downloaded from the JGI Phytozome 12.0 database (Available online: https://phytozome.jgi.doe.gov/pz/portal.html, accessed on 1 April 2021) [42] via the same method. 

The physico-chemical properties of molecular weight, and isoelectric points, were analyzed by the online ProtParam tool (Available online: http://web.expasy.org/protparam/, accessed on 1 April 2021) [43]. The subcellular localization of BnSOD proteins was predicted from the WoLF PSORT server (Available online: https://wolfpsort.hgc.jp/, accessed on 1 April 2021) [44]. *BnSOD* gene structures were constructed via TBtools software (V 1.068; https://github.com/CJ-Chen/TBtools, accessed on 1 April 2021) [45]. The conserved motifs about BnSOD protein sequences were identified using the MEME website (Available online: https://meme-suite.org/meme/db/motifs, accessed on 1 April 2021) [46]. 

### 2.2. Phylogenetic Tree and Synteny Analysis of BnSOD Proteins

To observe the evolutionary relationship of the *BnSOD* gene family, we constructed a phylogenetic tree about *B. napus, B. oleracea, B. rapa,* and *A. thaliana* protein sequences. The sequence alignment was performed by MEGA 7 software (Available online: https://megasoftware.net/home, accessed on 1 April 2021) [47]. The neighbor-joining (NJ) method was performed to construct a phylogenetic tree with 1000 bootstrap replicates using the Evolview v3 website (Available online: https://www.evolgenius.info/evolview, accessed on 1 April 2021) [48] to display the phylogenetic tree. Synteny relationships of *SOD* genes were developed by the python-package, JCVI (Available online: https://github.com/tanghaibao/jcvi, accessed on 1 April 2021) [49] from *B. napus, B. oleracea, B. rapa,* and *A. thaliana*. We calculated the Ka/Ks ratios of all *SODs* using KaKs_Calculator 2.0 (Available online: https://sourceforge.net/projects/kakscalculator2/, accessed on 1 April 2021) [50].

### 2.3. Analysis of Cis-Acting Regulatory Elements in the BnSODs Promoters 

To analyze the putative *cis*-elements in the *BnSODs* promoters, we extracted the 2Kb sequence upstream of start codons in the *B. napus* genome. Then, the promoter sequence of each gene was analyzed using the PlantCARE website (Available online: http://bioinformatics.psb.ugent.be/webtools/plantcare/html/, accessed on 1 April 2021) [51] and figures drawn using TBtools (V 1.068) [45]. 

### 2.4. Prediction of Putative miRNA Targeting BnSOD Genes and GO Annotation Analysis

The coding sequence (CDS) of *BnSODs* was used to identify possible target miRNAs in the psRNATarget database (Available online: http://plantgrn.noble.org/psRNATarget/, accessed on 01 April 2021) [52] with default parameters. We drew the interaction network figure between the miRNAs and *BnSOD* genes by Cytoscape software (V3.8.2; Available online: https://cytoscape.org/download.html, accessed on 1 April 2021). Gene ontology (GO) annotation analysis was performed by uploading all BnSODs protein sequences to the eggNOG website (Available online: http://eggnog-mapper.embl.de/, 1 April 2021) [53]. TBtools was used to perform GO enrichment analysis. 

### 2.5. Expression Profiling of BnSOD Genes in Different Tissues

For tissue-specific expression profiling, we downloaded RNA-seq data (BioProject ID: PRJCA001495) of rapeseed from the National Genomics Data Center. The complete method has been described in our recent work [37]. Briefly, Cuffquant and Cuffnorm were used to produce normalized counts in transcripts per million (TPM) values. Based on TPM values, the expression heat map was created using GraphPad Prism 8 software (https://www.graphpad.com/, accessed on 1 April 2021). [54].

### 2.6. Plant Material and Stress Conditions

In this study, a typical cultivated variety, “ZS11,” was used for stress treatments. The seeds of the “ZS11” genotype were provided by the OCRI-CAAS, Wuhan, China. The stress treatments were carried out as described in our recent work [37]. Briefly, the vigor seeds were cultivated on water-saturated filter paper in a chamber (25 °C day/night and 16 h/8 h light/dark cycle) till the radicle’s extent extended around 5 mm. For stress treatment, germinated seeds were subjected to 150 mM NaCl solution for salinity stress, 15% PEG6000 solution for drought stress, and 4 °C for cold stress on water-saturated filter paper. For waterlogging stress, the seeds were flooded with water in the Eppendorf tube. To evaluate the impact of diverse hormones, the germinated seeds were grown in Murashige and Skoog (MS) medium supplied with 100 μM abscisic acid (ABA), 100 μM gibberellic acid (GA), 100 μM indole-acetic acid/auxin (IAA), and 100 μM kinetin (KT). The samples were collected at 0 (CK), 2, 4, 6, and 8 h after the treatments. All of the treatments were performed with three biological replications. All of the samples were immediately frozen in liquid nitrogen and were stored at −80 °C for further experiments.

### 2.7. RNA Extraction and qRT-PCR Analysis

Total RNA extraction and cDNA synthesis were performed using a TransZol Up Plus RNA Kit (TransGen Biotech, Beijing, China) and cDNA Synthesis SuperMix (TransGen Biotech, Beijing, China) according to manufacturer instructions. The detailed information of qRT-PCR reactions has been described in our recently published work [37]. Initially, the expression data were analyzed using the 2^−ΔΔCT^ method. Due to the large difference in the expression levels, we used the log2 fold change method to calculate the expression results for better visualization of differently expressed genes under stress treatments. All of the primers used in this experiment are listed in Table S1. The heatmap was created using GraphPad Prism 8 software [54].

### 2.8. Prediction of the 3D Structure of BnSOD Proteins

The predicted 3D structures of BnSODs were created with the 3D LigandSite website (https://www.wass-michaelislab.org/3dlig/index.html, accessed on 1 April 2021) [55]. The probability score of the predicted sites shows the possibility of apiece residue to be elaborated in binding. During the 3D model predictions, we choose cluster 1 with a higher Z-score. The higher Z-score value indicates the reliability and trueness of the cluster/model [55].

## 3. Results

### 3.1. Identification of SOD Gene Family in B. napus

The current study identified 31 *BnSOD* genes in the rapeseed genome using eight *A. thaliana* (*AtSODs*) protein sequences as queries (Table 1; Table S2). According to the domain scrutiny, 14 proteins were found to have a Cu/Zn-SODs domain (Pfam: 00080), 11 proteins were found to have a Fe-SODs domain (Pfam: 00081), and six proteins were found to have an Mn-SODs domain (Pfam: 02777); hereafter, these genes were named as *BnCSD1-BnCSD14*, *BnFSD1-BnFSD11,* and *BnMSD1-BnMSD6*, respectively (Table 1). Comprehensive statistics of 31 *BnSOD* genes were documented in Table 1. Out of 31 *BnSODs*, 15 genes were located on the A subgenome, and 16 genes were located on the C subgenome (Table 1). The gene length extended from 826 bp (*BnCSD4*) to 6898 bp (*BnFSD5*) with 3–9 exons in the individual gene sequences. The CDS length, protein length, and molecular weight extended from 441–1173 bp, 146–390 amino acids, and 14.55–42.34 kDa (*BnCSD9-BnCSD2*), respectively. The isoelectric points extended from 4.88 (*BnFSD7*) to 9.56 (*BnMSD3*) (Table 1). The subcellular localization results prophesied that 15 proteins were located on the chloroplast, nine proteins were located on the cytoskeleton, five proteins were located on the mitochondrion, and the remaining two proteins (*BnFSD9* and *BnMSD6*) were located on the endoplasmic reticulum (Table 1).

Additionally, 14 SOD genes were also identified from *Brassica oleracea* (*BolCSD1-BolCSD6*, *BolFSD1-BolFSD5*, and *BolMSD1-BolMSD3*), and *Brassica rapa* (*BraCSD1-BraCSD7*, *BraFSD1-BraFSD4*, and *BraMSD1-BraMSD3*) genomes (Table S2).

### 3.2. Phylogenetic Relationships of SOD Genes

**Figure 1:**
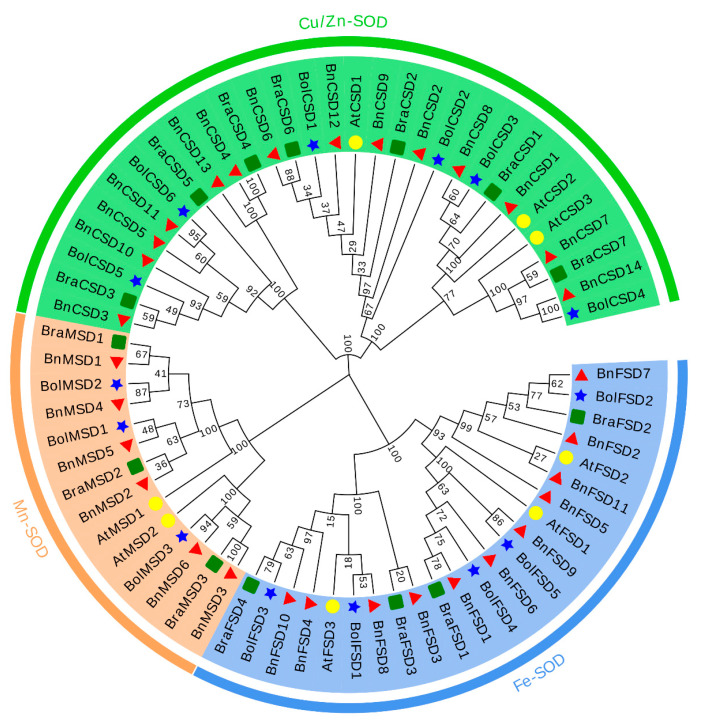
A neighbor-joining phylogenetic tree of 53 SOD proteins from *B. napus, B. oleracea, B. rapa,* and *A. thaliana*. Overall, 31 *BnSODs* (red triangle), 12 *BolSODs* (blue star), 12 *BraSODs* (green box), and 8 *AtSODs* (yellow circles) were grouped into three groups based on domain and 1000 bootstrap values. All of the notes indicate the percentage of bootstrap values.

In the current study, the evolutionary relationships were explored between *BnSODs*, *BolSODs, BraSODs,* and *AtSODs* genes. Based on domains (Cu/Zn-SODs, Fe-SODs, and Mn-SODs) and a phylogenetic tree, 67 *SODs* were clustered into three major groups (Figure 1). Results presented that the Cu/Zn-SODs group consists of 30 *SODs* members (14 *BnSODs*, 7 *BraSODs*, 6 *BolSODs,* and 3 *AtSODs*), the Mn-SODs group consists of 14 *SODs* members (6 *BnSODs*, 3 *BraSODs*, 3 *BolSODs,* and 2 *AtSODs*), and the Fe-SODs group consists of 23 *SODs* members (11 *BnSODs*, 4 *BraSODs*, 5 *BolSODs,* and 3 *AtSODs*) (Figure 1). Interestingly, Cu/Zn-SODs and Fe-SODs groups possessed a greater number of *SODs* than the Mn-SOD group. It was also found that the *BnSODs* exhibited a more closely phylogenetic relationship with *BolSODs* and *BraSODs* in each group.

### 3.3. Chromosomal Locations and Synteny Analysis of SOD Genes

**Figure 2:**
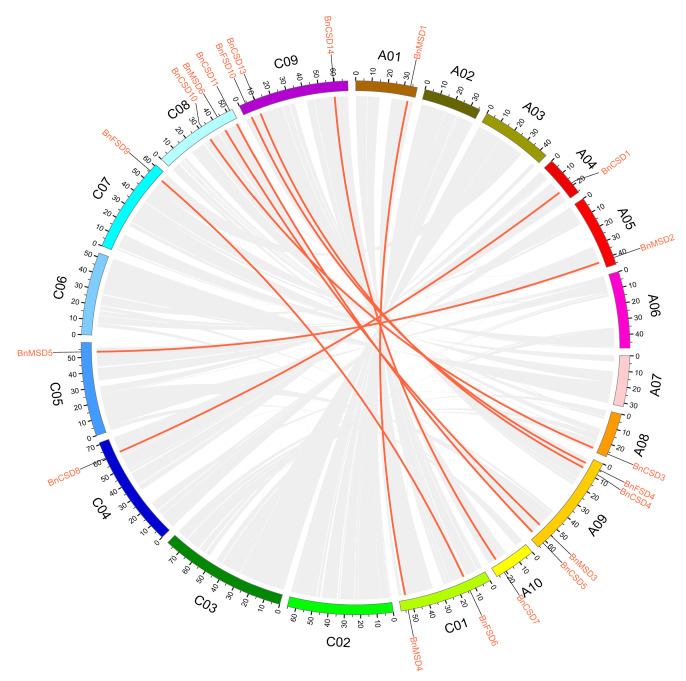
Chromosomal locations and inter-chromosomal associations of *BnSOD* genes. Grey lines in the background display all syntenic blocks in the *B. napus* genome, and the red lines display syntenic *BnSODs* gene pairs.

Gene duplications (tandem and segmental) are considered the main driving forces in promoting new genomic evolution [56]. Thus, gene duplication events were evaluated between *BnSODs, BolSODs, BraSODs*, and *AtSODs* (Table S3). The chromosol location of 10 *BnSODs* gene pairs was examined (Figure 2). Twelve out of 19 chromosomes harbored *BnSODs*. Particularly, chromosomes A01, A04, A05, A08, A10, C04, C05, and C07 possessed one gene, chromosome C01 contained two genes, chromosomes C08 and C09 possessed three genes, and chromosome A09 contained four genes (Figure 2). Surprisingly, the residual chromosomes did not comprehend *BnSOD* genes. Our results show that segmental duplications have played vital parts in developing *BnSOD* genes in the rapeseed genome (Table S3). Moreover, no tandem duplication events were detected. Notably, a gene pair (*BnMSD6* and *BnMSD6*) was found to be dispersed, while the remaining gene pairs experienced segmental duplications (Table S3).

**Figure 3:**
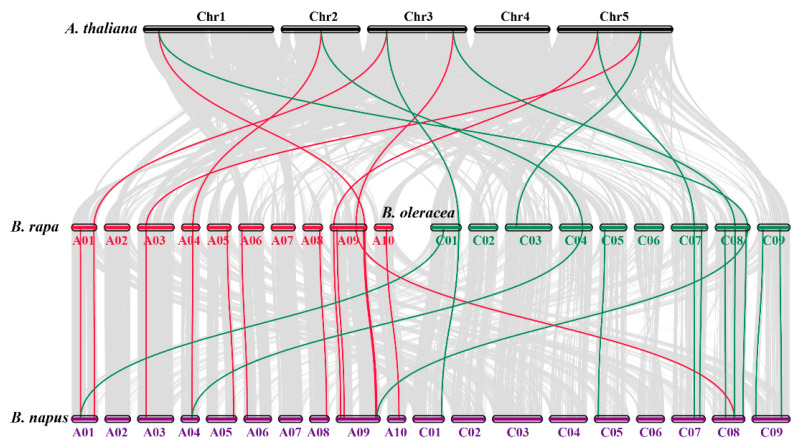
Collinearity analysis of SOD genes in *B. napus, B. rapa, B. oleracea,* and *A. thaliana* chromosomes. Grey lines in the background show the collinear blocks within *B. napus* and other plant genomes, whereas the red and green lines represent the syntenic SOD gene pairs. Genes located on the *B. napus* A subgenome are syntenic with *B. rapa, B. oleracea,* and *A. thaliana*, whereas genes located on *B. napus* C subgenome are mainly syntenic with *B. oleracea* and *A. thaliana* except for one gene that showed a syntenic relationship with *B. rapa* and *A. thaliana*.

Collinearity investigation revealed strong orthologs of *SOD* genes among *B*. *napus* and three closely related species (*B. rapa, B. oleracea,* and *A. thaliana*) (Figure 3). In summary, in the A subgenome, seven and five *B. napus* genes demonstrated syntenic relations with *BraSODs* and *AtSODs,* respectively. Three genes exhibited syntenic relations with *BolSODs* and *AtSODs* (Figure 3). In contrast, in the C subgenome, six and three *B. napus* genes displayed syntenic relations with *BolSODs* and *AtSODs.* One gene revealed syntenic relations with *BnSODs* and *AtSODs* (Figure 3). Our results revealed numerous *A. thaliana*, *B*. *rapa*, and *B*. *oleracea* homologous sustained syntenic relations with *BnSODs*, suggesting that whole-genome duplication or segmental duplications played a significant part in *BnSODs* gene family progression (Table S3).

The Ka/Ks ratio is considered a significant index in evaluating the duplication events and selection pressures [57]. Therefore, to understand the evolutionary story of the *BnSODs*, the Ka, Ks, and Ka/Ks ratio was calculated for *B. napus* and the other three plant species (Table S3). Our results revealed that all of the duplicated *BnSOD* gene pairs had a Ka/Ks ratio of <1 (Table S3), signifying that the *BnSOD* genes might have handled intense purifying selective pressure through their evolution (Table S3). Similar findings were observed in *B. rapa, B. oleracea,* and *A. thaliana* (Table S3).

### 3.4. BnSOD Gene Structures and Conserved Motifs Investigation

**Figure 4:**
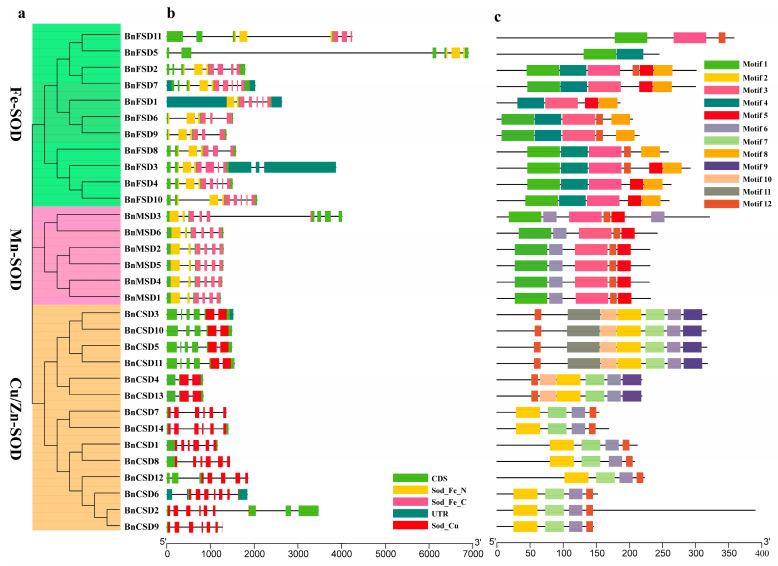
The gene structure and motif analysis of *BnSODs*. (**a**) Based on phylogenetic relationships and domain identification, the *BnSODs* were grouped into three groups. (**b**) Gene structure of *BnSODs.* The blue-gray color represents UTR regions, the light green color represents CDS or exons, the yellow color represents Sod_Fe_N, the pink color represents Sod_Fe_C, the red color represents Sod_Cu, and the black horizontal line represents introns. (**c**) Conserved motif compositions identified in *BnSODs*. Different color boxes denote different motifs.

The exons–introns structures and the introns number play a vital role in gene family evolution [12,58]. The comprehensive examination of the phylogenetic relationships and gene structure illustration supported our knowledge of *BnSODs* gene structures (Figure 4). The findings exposed that exon and intron numbers of *BnSOD* genes had high inconsistency and were steady with the evolutionary hierarchy outcomes, i.e., the numbers of introns and exons were found to be relatively similar within the same group (Figure 4a,b). Briefly, the number of exons and introns varied from three to nine and two to eight in the individual gene sequences, respectively (Table 1; Figure 4b). A group with the Fe-SOD domain had five–nine exons and four–eight introns. A group with the Mn-SOD domain had six exons and five introns (five genes), and only one gene (*BnMSD3*) had nine exons and eight introns. Whereas, the group with Cu/Zn-SOD had five–nine exons and two–eight introns (Table 1; Figure 4b). Notably, group Fe-SOD and Cu/Zn-SOD groups have complex structures compared to the Mn-SOD group.

To systematically appraise BnSODs’ protein structure diversity and envisage their functions, we explored the full-length protein sequences of 31 BnSODs by MEME software to identify their conserved motifs. The investigation outcomes displayed that a total of 12 conserved motifs were found (Figure 4c). The detailed information (name, sequence, width, and E-value) of identified motifs is presented in Table S4. In short, the conserved motifs of SOD proteins varied from two to seven, and the motifs distributions were in agreement with the groups. In the Fe-SOD group, only two proteins (BnFSD11 and BnFSD5) had three and two motifs, respectively (Figure 4c). Interestingly, motifs 1, 3, 4, 5, and 8 were predicted to be specific to Fe-SOD and Mn-SOD groups. Motif 6 was specific to Mn-SOD and Cu/Zn groups. Motifs 2, 9, 10, and 11 were specific to the Cu/Zn group. Only motif 12 was largely distributed among all of the domain groups (Figure 4c). In summary, the group arrangements’ reliability was mightily sustained by investigating conserved motif arrangements, gene structures, and phylogenetic relationships, suggesting that BnSOD proteins have tremendously well-maintained amino acid remains and members within a group. Thus, it can be assumed that proteins with similar structures and motifs might play similar functional roles.

### 3.5. Examination of Cis-Elements in Promoters of BnSOD Genes

**Figure 5:**
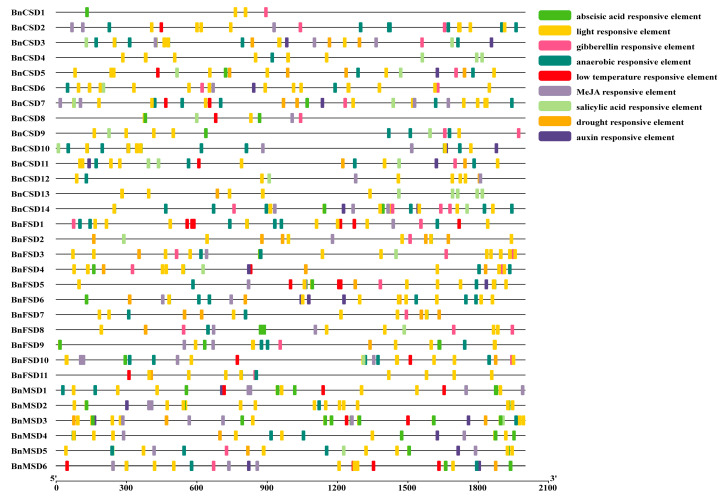
Analysis of *cis*-regulatory elements in the *BnSODs* promoter regions. Different *cis*-elements with functional similarity are denoted by similar colors.

To distinguish the gene functions and regulatory roles, *cis*-regulatory elements in *BnSODs* promoter regions were examined by searching a 2000 bp upstream region from each gene’s transcriptional activation site against the PlantCARE database. The detailed information of *cis*-elements is presented in Table S5. As revealed in Figure 5, five phytohormone-correlated [(abscisic acid (ABA), auxin, methyl jasmonate (MeJA), gibberellin (GA), and salicylic acid (SA)] responsive elements including TCA-element, CGTCA-motif, ABRE, TGACG-motif, TATC-box, GARE-motif, P-box, etc. were recognized (Figure 5; Table S5). Particularly, numerous phytohormone-correlated elements were predicted to be specific to some genes and widely distributed (Figure 5), signifying the crucial role of these genes in phytohormone-mediation.

Furthermore, four stress-responsive (drought, low-temperature, anaerobic, and light) elements, including ARE, LAMP-element, LTR, TCT-motif, chs-CMA1a, MBS, G-box, GT1-motif, MBS, etc. were identified (Figure 5; Table S5). Mainly, numerous light-responsive elements were found to be widely distributed among all of the genes (Figure 5; Table S5), signifying the substantial role of *BnSODs* in response to light stress. Overall, results advised that *BnSODs* expression levels may diverge under phytohormone and abiotic stress conditions.

### 3.6. Genome-Wide Analysis of miRNA Targeting BnSOD Genes

**Figure 6:**
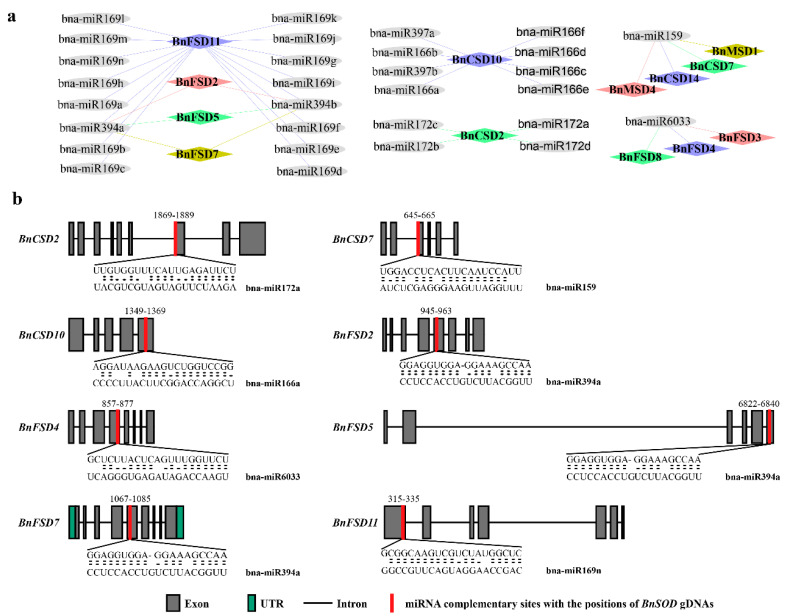
miRNA targeting *BnSOD* genes. (**a**) Network diagram of predicted miRNA targeting *BnSODs* genes. Different diamond colors represent *BnSODs* genes, and gray ellipse shapes represent miRNAs. (**b**) Schematic illustration indicates the *BnSODs* targeted by miRNAs. The RNA sequence of each complementary site from 5’-3’ and the predicted miRNA sequence from 3’-5’ are exposed in the long-drawn-out areas. See Table S6 for the detailed data of all predicted miRNAs.

In the recent past, several studies have revealed that miRNA-mediated regulation accompanies the stress responses in plants. Thus, for a deep understanding of miRNA-mediated post-transcriptional regulation of *BnSODs*, we predicted 30 miRNAs targeting 13 genes (Figure 6a; Table S6). Some of the miRNA-targeted sites are presented in Figure 6b, whereas the detailed information of all miRNAs targeted genes/sites is presented in Table S6. Our results showed that four members of the bna-miR159 family targeted four genes (*BnCSD7, BnCSD14*, *BnMSD1*, and *BnMSD4*). Six members of the bna-miR166 family targeted one gene (*BnCSD10*). Fourteen members of the bna-miR169 family targeted one gene (*BnFSD11*). Four members of the bna-miR172 family targeted one gene (*BnCSD2*). Two members of the bna-miR394 family targeted four genes (*BnFSD11, BnFSD2, BnFSD7,* and *BnFSD5*). Two members of the bna-miR397 family targeted one gene (*BnCSD10*). One member of the bna-miR6033 family targeted three genes (*BnFSD4, BnFSD8,* and *BnFSD3*) (Figure 6a; Table S6). Mainly, *BnFSD11, BnCSD10*, and *BnCSD2* were prophesied to be targeted by a greater number of miRNAs (Figure 6a; Table S6). The expression levels of these miRNAs and their targeted genes requires validation in additional research to govern their biological roles in the rapeseed genome.

### 3.7. Functional Annotation Study of BnSOD Genes

The functions of the *BnSODs* genes were prophesied through GO annotation investigation based on biological process (BP), molecular function (MF), and cellular component (CC) classes. The GO annotation results revealed numerous significantly enriched terms (Table S7). Analysis of BP annotations indicated that these genes were mainly involved in responses to stimulus (GO:0050896), responses to chemicals (GO:0042221), responses to stress (GO:0006950), responses to inorganic substances (GO:0010035), responses to abiotic stimulus (GO:0009628), cellular oxidant detoxification (GO:0098869), cellular response to oxygen-containing compound (GO:1901701), etc. (Table S7). Analysis of MF annotations revealed that these genes were primarily involved in ion binding (GO:0043167), copper ion binding (GO:0005507), zinc ion binding (GO:0008270), oxidoreductase activity (GO:0016491), superoxide dismutase activity (GO:0004784), antioxidant activity (GO:0016209), etc. (Table S7). A study of CC annotations showed that these genes are mainly involved in cellular anatomical entity (GO:0110165), cytoplasm (GO:0005737), obsolete intracellular part (GO:0044424), cellular component (GO:0005575), intracellular anatomical structure (GO:0005622), membrane-bounded organelle (GO:0043227), etc. (Table S7). In conclusion, GO annotation results confirmed the *BnSODs* role in response to different stress stimulus, cellular oxidant detoxification processes, metal ion binding activities, SOD activity, and different cellular components.

### 3.8. Expression Analysis of BnSOD Genes in Several Developmental Tissues

**Figure 7:**
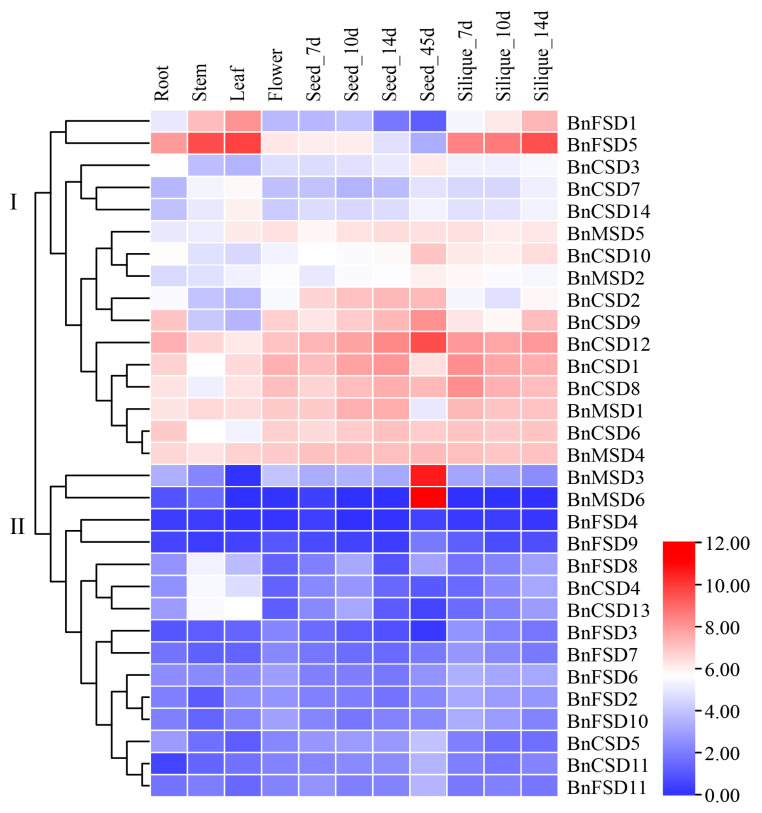
Expression analysis of *BnSOD* genes in several developmental organs. The 7d, 10d, 14d, and 45d tags indicate the time-points when the samples were harvested. In the expression bar, the red color shows high, and the blue color shows low, expression levels. The expression heat map was created using transcripts per million (TPM) values.

Tissue-specific expression levels of *BnSODs* genes were appraised in six different tissues and organs (including roots, stems, leaves, flowers, seeds, and silique) using RNA-seq data from *B. napus* (ZS11 variety) (BioProject ID: PRJCA001495). As shown in Figure 7, the group I genes display higher expression in all of the tissues except *BnFSD1, BnCSD3, BnCSD7, BnCSD14, BnMSD2,* that showed relatively lower expression than other genes (Figure 7). Meanwhile, genes clustered in group II exhibited considerably low expression, whereas *BnMSD3* and *BnMSD6* showed high expression at the seed_45d stage (Figure 7). Overall, results showed that genes from group I may play significant roles in *B. napus* growth and development.

### 3.9. Expression Profiles of BnSOD Genes under Hormones and Abiotic Stress Treatments

**Figure 8:**
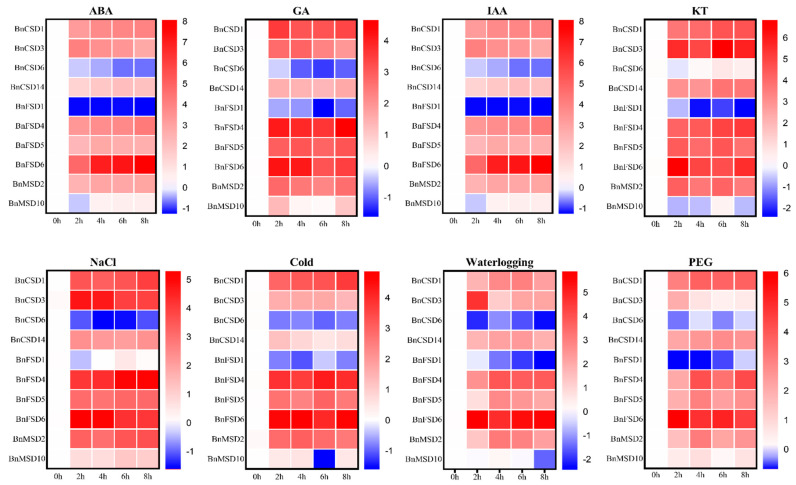
*BnSOD* gene expression levels under different hormone and abiotic stress conditions at different time points (0 (CK), 2, 4, 6, and 8 h). The expression bars display the comparative gene expression trends based on the log2 fold change values. In the expression bar, the red color displays high, and the blue color displays low, expression levels.

To examine the *BnSOD* genes’ expression levels under different hormones (ABA, GA, IAA, and KT) and abiotic stress (salinity, cold, waterlogging, and drought) treatments, the qRT-PCR analysis of 10 randomly selected *BnSOD* genes was applied to govern the transcription profile (Figure 8). Under all of these stress conditions, *BnCSD6* and *BnFSD1* were down-regulated and showed relatively low expression levels. Likewise, *BnMSD10* showed a considerable response to ABA, GA, IAA, salinity, cold, and drought stress at different time points. The other eight genes were significantly up-regulated and showed higher expression to all hormones and abiotic treatments (Figure 8), signifying that these genes may play a significant role in mitigating various hormone and abiotic stresses.

### 3.10. Prediction of the 3D Structures of BnSODs

**Figure 9:**
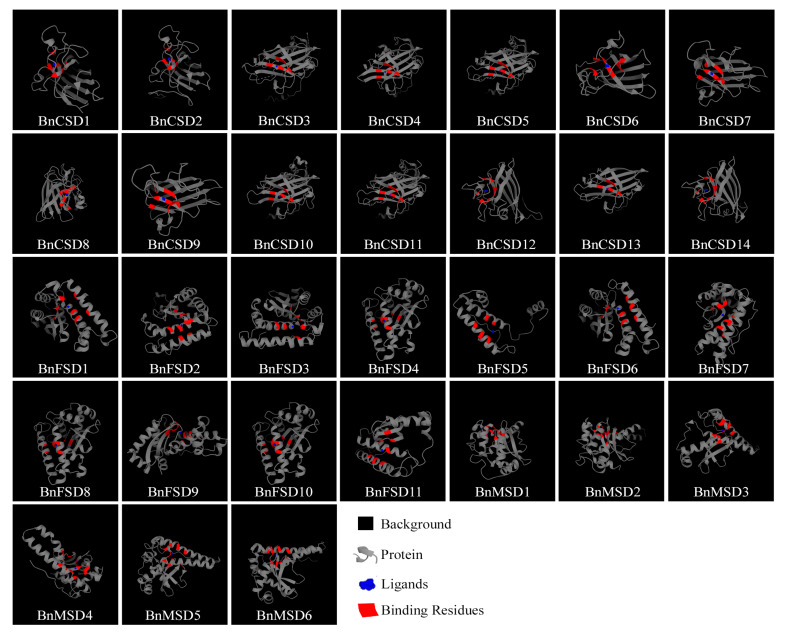
Predicted 3D structures and binding sites of BnSODs proteins using a 3DLigandSite tool. See Table 1 for the complete protein information, including subcellular location. See Table S8 for the complete information of the predicted 3D models, including predicted binding sites, conserved residues, and probability levels.

The 3D structures of BnSODs proteins were predicted using the 3DLigandSite tool with the default Search probability threshold (80.0%). The created models were downloaded to view the 3D structures (Figure 9). The detailed information of predicted 3D structures is presented in Table S8. The predicted Cu/Zn-SODs (BnCSD1-BnCSD14) model possessed a primarily highly conserved β-barrel structure, including a few short α-helices (Figure 9). The distinctive quaternary model of eukaryotic Cu/ZnSODs proteins contained a β-barrel domain comprised of eight antiparallel β-strands and Cu/Zn binding sites positioned at the exterior of the β-barrel in the active site network [13]. An individual subunit of Cu/ZnSODs proteins, the binding site possessed the Cu/Zn ion ligated by four histidines and important enzymes (such as arginine and alanine) with different conserved residues (Table S8). The conserved disulfide bond residues were examined in the active site channel of Cu/ZnSODs, including Cys202, Cys110, Cys201, and Cys110 in BnCSD3, BnCSD4, BnCSD10, BnCSD13, respectively (Table S8). The remaining Cu/ZnSODs proteins do not possess the Cys–Cys conserved disulfide bonds.

Likewise, the examination of *B. napus* Fe/MnSODs discovered that the dominant α-helices and β-sheet structures were detected (Figure 9). However, considerable variance in the sum of α-helices was observed and missing the disulfide bonds (exclusive features of Cu/ZnSODs). For instance, the active spot of Mn/FeSODs was positioned among the N- and C-terminal domains, and it varied from that of Cu/ZnSODs as it was comprised of a single metal ion. The metal ion is harmonized in a stressed trigonal bipyramidal geometry through eight–ten amino acid side-chains (including HIS, HIS, TYR, TRP, GLN, ASP, ALA) (Table S8) with solvent molecules. These Mn/FeSODs active sites also comprise hydrogen bonds that spread from the metal-bound solvent molecule to solvent-visible residues at the edge among subunits (Table S8). Additional structural investigation of these amino acids can boost our understanding of the catalytic process and metal ion binding.

## 4. Discussion

Rapeseed is an allotetraploid crop that experiences extensive genome repetition and integration actions [59]. Nonetheless, rapeseed yield is affected by several abiotic stresses, including cold, heat, salinity, drought, and heavy metals [33,34]. ROS production under typical and stress environments is measured and scavenged through SODs, which act as the primary markers of an enzyme complicated in ROS scavenging, and play a crucial role in plants’ physiological and biochemical procedures to survive with environmental cues [3]. Over the last few years, *SOD* family genes have been documented in different plant species, such as five *SOD* genes in *Zostera marina* [60], seven genes in *Medicago truncatula* [12] and barley [61], eight genes in sorghum [62], nine genes in tomato [63], ten genes in grapevine [64], 18 genes in cotton [28], 25 genes in banana [65], 26 genes in wheat [66], and 29 genes in *B. juncea* [67]. Thus far, there is no wide-ranging investigation of the *SOD* family genes in rapeseed. The obtainability of the whole rapeseed genome permits the genome-wide characterization of the SOD family genes, which may be used for future rapeseed improvement.

In the current study, we identified 31 *BnSOD* genes, including 14 *Cu/Zn-SODs*, 11 *Fe-SODs*, and six *Mn-SODs* genes (Table 1), which were clustered into three major sets based on the binding domain (Figure 1). So far, this is the largest *SOD* gene family identified in the rapeseed genome. Changes in the SOD gene numbers among plant species may be accredited to gene repetition events, including tandem and segmental repetitions, and act as a key factor in extending *SODs* for divergence. Gene doubling of SOD genes was also detected in various plant species [67,68]. Our results showed that *BnSODs* had experienced segmental duplications (Table S3). Consequently, these outcomes suggested that *BnSODs* duplication actions might play an important role in gene progression.

Gene structure analysis discovered that the number of exons varied from three to nine, and the number of introns varied from two to eight (Table 1; Figure 4). The Fe-SOD group had five to nine exons and four to eight introns. The Mn-SOD group had six exons and five introns (five genes), and only one gene (*BnMSD3*) had nine exons and eight introns. In comparison, the group with Cu/Zn-SOD had five to nine exons and two to eight introns (Table 1; Figure 4b). In wheat, seven *TaSODs* genes were found to have seven introns [66]. In sorghum, intron numbers varied from five to seven [62]. The exon–intron assemblies’ difference was proficient by three critical apparatuses (exon/intron gain/loss, exonization/pseudoexonizationand, insertion/deletion), and they are directly subsidized to structural discrepancy [62,69]. Interestingly, the *SOD* genes in each cluster presented similar exon–intron association and conserved motifs (Figure 4), signifying that these genes may participate in the same roles connected to numerous abiotic stressors. Comparable findings have also been reported in sorghum [62], tomato [63], cotton [28], and wheat [66].

To better understand the *BnSOD* genes’ role against several environmental strains, *cis*-elements in the promoter regions were prophesied. Our results exhibited that three types of *cis*-elements were recognized: stress-, hormones-, and light-responsive (Figure 5; Table S5). Most of the identified *cis*-elements were connected with ABA, MeJA, GA, SA, drought, low-temperature, light, and anaerobic induction. According to previous reports, *cis*-elements subsidize to plant stress responses [70,71]. These consequences were further validated by the GO annotation analysis (Table S7). Moreover, several researchers reported similar findings in different crop plants where *SOD* genes were found to play a significant part under various stress conditions [62,66,67]. These outcomes can boost our understanding of *BnSOD* genes under diverse environmental conditions.

Previous investigations have described that *SOD* genes may show diverse expression levels in tissues and under stress conditions [62,63,72]. Thus, in the current study, tissue-specific expression levels of *BnSODs* genes were appraised in six different developmental tissues using RNA-seq data (Figure 7), which were in agreement with earlier findings [12,63,66]. Several genes displayed higher expression in all evaluated tissues, indicating that these genes may contribute to rapeseed growth and development. Similarly, the expression levels of 10 *BnSOD* genes were evaluated under different hormones and abiotic stress treatments (Figure 8). Almost all genes were up-regulated under stress treatments, excluding *BnCSD6* and *BnFSD1* genes, whose expression levels were down-regulated. These results agreed with previous findings where several *SOD* genes showed higher expression in response to stresses. For instance, in grapevines, many *SODs* were up-regulated under cold, heat, salinity, and drought treatment [72]. All *SODs* were significantly up-regulated in tomatoes under salt and drought stress [63]. In wheat, almost all *SODs* showed higher expression in response to mannitol, salinity, and drought stress than control conditions [66]. Under cold stress conditions, SOD activity was significantly increased in rapeseed [34]. These findings provided strong evidence that *SOD* genes play a conserved role in vindicating abiotic stresses in different plant species.

Over the past few years, abundant miRNAs have been recognized through genome-wide examination in rapeseed to participate in diverse environmental factors [73,74,75,76,77]. In the present study, we identified 30 miRNAs from seven families targeting 13 *BnSODs* genes (Figure 6a; Table S6). In a recent study, 20 miRNAs were predicted to target 14 *SOD* genes in cotton [28]. Previously, miR398 targeted two Cu/ZnSOD genes in *Arabidopsis thaliana* [27]. miR166 has been stated to be pointedly up-regulated against UV-B radiation in maize [78]; in cassava against cold and drought stresses [79]; in Chinese cabbage under heat stress conditions [79]. According to recent reports, bna-miR159 plays a vital role in fatty acid metabolism in rapeseed [80,81]. Several members of miR172 have been detected while participating in plant development, including regulation of flowering time and floral patterning [82], and developmental control in *A. thaliana* [83]. miR394 has been reported to be involved in drought and salinity tolerance in *A. thaliana* via an ABA-dependent pathway [84]. In another study, miR394 was associated with cold stress responses in *A. thaliana* [85]. Shortly, these reports support our results and recommend that bna-miRNAs might play pivotal roles against several stresses by altering the transcript levels of *SOD* genes in rapeseed.

To obtain further insight, we also predicted the 3D structures of BnSODs proteins (Figure 9). The Cu/Zn-SODs (BnCSD1-BnCSD14) models had primarily highly conserved β-barrel structures, including a few short α-helices (Figure 9). According to a recent report, eukaryotic Cu/ZnSODs proteins contain a β-barrel domain comprised of eight antiparallel β-strands and Cu/Zn binding sites positioned at the exterior of the β-barrel in the active site network [13]. In Cu/ZnSODs proteins, the binding site possessed Cu/Zn ions ligated by four histidines and important enzymes (such as arginine and alanine) with different conserved residues (Table S8). These results were in agreement with previously predicted 3D models of SODs in sorghum [62], in *Gossypium raimondii,* and *G. arboretum* [86], and in rice [87]. The previous study proved that the binding active site of metal ions and the generation of the conserved disulfide bond in individual subunits would participate in the protein constancy, specificity, and dimer gathering [62,88]. Similarly, conserved residues (including arginine residue) also supported our results, and these residues were considered to be vital players for normal enzymatic movement (Table S8) [88]. Similarly, the Fe/MnSODs models of rapeseed suggested that the β-sheets were conquered by α-helices (Figure 9), and these results were in agreement with recently predicted SOD models in rice [87], soybean [89], and sorghum [62].

## 5. Conclusions

In the current study, we identified 31 *BnSOD* genes in the rapeseed genome via genome-wide comprehensive investigation. To boost our understanding, gene structure, phylogenetic and synteny, conserved motifs, *cis*-elements, GO annotation, miRNA prediction, 3D protein structure, tissue-specific expression, and expression profiling under different hormones and abiotic stress treatments have been performed. The results showed that some genes significantly responded to both hormone and abiotic stress stimuli, enhancing our understanding of the *BnSOD* genes. Thus, additional investigations are required to confirm the purposeful role of *BnSOD* genes in rapeseed growth, development, and response to numerous environmental cues.

## Figures and Tables

**Table 1:** The data of 31 *SOD* genes identified in rapeseed genome.

Gene ID	Gene Name	Genomic Position (bp)	Gene Length (bp)	CDS Length (bp)	Exons	Protein Length (Amino Acids)	Molecular Weight (kDa)	Isoelectric Point (pI)	Subcellular Localization
BnaA04T0182200ZS	*BnCSD1*	A04-18599782:18600942 (+)	1160	639	6	212	22.15	7.16	Chloroplast
BnaA06T0053200ZS	*BnCSD2*	A06-3364923:3368393 (+)	3470	1173	9	390	42.34	8.71	Cytoskeleton
BnaA08T0278700ZS	*BnCSD3*	A08-26336887:26338407 (+)	1520	954	6	317	33.76	5.19	Chloroplast
BnaA09T0129500ZS	*BnCSD4*	A09-7757584:7758410 (−)	826	660	3	219	23.60	5.95	Chloroplast
BnaA09T0647100ZS	*BnCSD5*	A09-61965958:61967448 (+)	1490	954	6	317	33.45	5.26	Chloroplast
BnaA09T0664700ZS	*BnCSD6*	A09-62938095:62939938 (−)	1843	459	8	152	15.17	5.64	Cytoskeleton
BnaA10T0190600ZS	*BnCSD7*	A01-21329437:21330797 (−)	1360	465	6	154	15.94	7.12	Cytoskeleton
BnaC04T0482300ZS	*BnCSD8*	C04-60854831:60856275 (+)	1444	627	6	208	21.60	7.84	Chloroplast
BnaC05T0066000ZS	*BnCSD9*	C05-3767908:3769181 (+)	1273	441	7	146	14.55	5.44	Cytoskeleton
BnaC08T0217500ZS	*BnCSD10*	C08-32001876:32003366 (−)	1490	951	5	316	33.71	6.91	Chloroplast
BnaC08T0505400ZS	*BnCSD11*	C08-51947641:51949188 (+)	1547	957	6	318	33.58	4.97	Chloroplast
BnaC08T0529200ZS	*BnCSD12*	C08-53323394:53325251 (−)	1857	672	7	223	23.42	6.66	Cytoskeleton
BnaC09T0137500ZS	*BnCSD13*	C09-10172656:10173492 (−)	836	660	3	219	23.72	6.83	Chloroplast
BnaC09T0484500ZS	*BnCSD14*	C09-59599637:59601046 (−)	1409	510	6	169	17.63	6.82	Cytoskeleton
BnaA01T0146300ZS	*BnFSD1*	A01-8654405:8657035 (−)	2630	561	6	186	21.12	6.06	Cytoskeleton
BnaA03T0141400ZS	*BnFSD2*	A03-7188761:7190553 (+)	1792	906	8	301	34.51	4.97	Chloroplast
BnaA06T0320600ZS.1	*BnFSD3*	A06-40740605:40744473 (+)	3868	879	9	292	33.60	7.58	Chloroplast
BnaA09T0072700ZS	*BnFSD4*	A09-4315462:4316968 (−)	1506	792	8	263	30.16	7.76	Chloroplast
BnaA10T0083700ZS	*BnFSD5*	A10-13303579:13310477 (−)	6898	738	6	245	28.02	9.44	Mitochondrion
BnaC01T0186100ZS	*BnFSD6*	C01-13777631:13779146 (−)	1515	618	5	205	22.99	6.3	Cytoskeleton
BnaC03T0164000ZS	*BnFSD7*	C03-9109698:9111720 (+)	2022	903	9	300	34.39	4.88	Chloroplast
BnaC07T0373300ZS	*BnFSD8*	C07-49894566:49896148 (−)	1582	780	6	259	29.58	8.58	Chloroplast
BnaC07T0462000ZS	*BnFSD9*	C07-55868729:55870093 (−)	1364	648	5	215	24.17	6.65	Endoplasmic reticulum
BnaC09T0062700ZS	*BnFSD10*	C09-4091989:4094054 (−)	2065	783	8	260	29.72	6.66	Chloroplast
BnaC09T0329400ZS	*BnFSD11*	C09-41111988:41116227 (−)	4239	1077	7	358	40.40	8.8	Chloroplast
BnaA01T0376200ZS	*BnMSD1*	A01-33774266:33775498 (−)	1232	699	6	232	25.50	8.38	Mitochondrion
BnaA05T0446100ZS	*BnMSD2*	A05-41549634:41550932 (−)	1298	696	6	231	25.41	8.47	Mitochondrion
BnaA09T0519400ZS	*BnMSD3*	A09-55258451:55262461 (−)	4010	966	9	321	35.60	9.56	Cytoskeleton
BnaC01T0471300ZS	*BnMSD4*	C01-53548352:53549620 (−)	1268	693	6	230	25.36	8.94	Mitochondrion
BnaC05T0492700ZS	*BnMSD5*	C05-53531802:53533097 (−)	1295	696	6	231	25.43	7.83	Mitochondrion
BnaC08T0362400ZS	*BnMSD6*	C08-43431847:43433140 (−)	1293	729	6	242	27.11	6.14	Endoplasmic reticulum

In the genomic position, the positive (+) and negative (−) sign shows the presence of a gene on the positive and negative strand of that specific marker correspondingly. CSD means Cu/Zn-SOD; FSD means Fe-SOD; and MSD means Mn-SOD.

## Data Availability

The datasets used and/or analyzed during the current study are shown in the supplementary files. For tissue-specific expression profiling, we downloaded RNA-seq data of rapeseed (BioProject ID: PRJCA001495) from the National Genomics Data Center. The rapeseed genome sequence was downloaded from BnPIR database (http://cbi.hzau.edu.cn/bnapus/index.php, accessed on 1 April 2021). The sequences of *Brassica rapa* and *Brassica oleracea* are available at Phytozome database (https://phytozome.jgi.doe.gov/pz/portal.html, accessed on 1 April 2021).

## Supplementary Materials

The following are available online at https://www.mdpi.com/article/10.3390/antiox10081182/s1, Table S1: the list primer was used for gene expression analysis by qRT-PCR, Table S2: the protein sequences of SOD family genes in *Arabidopsis thaliana, Brassica napus, Brassica oleracea,* and *Brassica rapa*, Table S3: results of segmentally and tandemly duplicated relationships, and Ka, Ks, and Ka/Ks ratio values between *B. napus, B. rapa, B. oleracea,* and *A. thaliana* gene pairs, Table S4: the information of identified 12 motifs in BnSOD proteins, Table S5: information of hormone- and stress-related *cis*-elements detected in the promoter regions of BnSODs, Table S6: information of predicted miRNA targeting *BnSOD* genes, Table S7: the GO enrichment analysis of *BnSOD* genes, Table S8: the specifics of predicted BnSODs 3D structures including predicted binding sites, conserved residues, and probability level.
